# Prelimbic of Medial Prefrontal Cortex GABA Modulation through Testosterone on Spatial Learning and Memory

**DOI:** 10.22037/ijpr.2019.1100745

**Published:** 2019

**Authors:** Azadeh Gholaminejad, Hamid Gholamipour-Badie, Mohammad Nasehi, Nasser Naghdi

**Affiliations:** a *Department of Physiology and Pharmacology, Pasteur Institute of Iran (IPI), Tehran, Iran.*; b *Department of Cognitive Neuroscience, Institute for Cognitive Science Studies (ICSS), Tehran, Iran.*; c *Cognitive and neuroscience research center (CNRC), Tehran Medical Sciences, Islamic Azad University, Tehran, Iran.*

**Keywords:** Prelimbic, Spatial memory, Testosterone, Bicuculline, GABA_A_ receptor, Morris Water Maze

## Abstract

Prefrontal cortex (PFC) is involved in multiple functions including attentional processes, spatial orientation, short-term memory, and long-term memory. Our previous study indicated that microinjection of testosterone in CA1 impaired spatial learning and memory. Some evidence suggests that impairment effect of testosterone is mediated by GABAergic system. In the present study, we investigated the interaction of testosterone (androgenic receptor agonist) and bicuculline (GABA_A_ receptor antagonist) on spatial learning and memory performance in the prelimbic (PL) of male Wistar rats. Cannulae were bilaterally implanted into the PL region of PFC and drugs were daily microinjected for two minutes in each side. There are 4 experiments. In the first experiment, three sham groups were operated (solvent of testosterone, bicuculline, testosterone plus bicuculline). In the second experiment, different doses of testosterone (40, 80 μg /0.5 μL DMSO/each side) were injected into the PL before each session. In the third experiment, intra PL injections of bicuculline (2, 4 μg/0.5 μL DMSO/each side) were given before every session. In the last experiment, testosterone (80μg/0.5 μL DMSO/each side) along with bicuculline (2 μg/0.5 μL DMSO/each side) was injected into the PL. The results showed there is no difference between control group and sham operated group. Testosterone 80 μg and bicuculline 2 μg, each given separately, and also in combination increased escape latency to find the platform compared to the sham operated and cause to impaired spatial learning and memory. It is shown that intra PL microinjection of bicuculline after testosterone treatment could not rescue the spatial learning and memory impaired induced by testosterone.

## Introduction

Prefrontal cortex has been proposed to be involved in multiple functions including working memory, temporary storage of information, attentional processes, and long-term memory and memory consolidation (1). Experimental and clinical evidence suggest that PFC damage leads to a spectrum of cognitive impairments including problems of attention, spatial orientation, short-term memory, temporal and source memory, meta memory, associative learning, creativity, perseveration, and reasoning (2). Also, several studies have demonstrated that the medial prefrontal cortex (mPFC) has an important role in learning and memory (3). Previous data emphasize the involvement of mPFC in the consolidation and retrieval of recently acquired spatial memories (4). Based on previous research mPFC is involved in spatial information processing, similar to the hippocampus. There is a role of the mPFC in spatial information processing from results obtained using maze procedures (Morris water maze) which primarily rely on reference memory (5). Rats with permanent or transient lesions of the medial prefrontal cortex (prelimbic/infralimbic) are impaired in switching from spatial-to visual-cued versions of the Morris water maze (6). Also it has been shown that while inactivation of mPFC leaves recent memory intact, it results in impairment of remote memory retrieval in a diverse range of tasks including the radial arm maze and the Morris Water Maze (7). Prelimbic as a part of mPFC, shows its best role in a particular kind of processing including maintaining information across a delay or manipulating such information to select a response and in processing certain types of information such as spatial vs. nonspatial information. GABA transmission has been proposed to facilitate the direction of attentional resources, enabling accurate encoding of information within working memory in the intact PFC (8). It has been reported that neurosteroid synthesis occurs in various regions of the brain such as cortex, hippocampus, and amygdale (9). Studies in humans and animal models have shown that in both males and females, gonadal hormones are capable of modulating PFC function (10). Some metabolites of pregnenolone, progesterone, testosterone, and deoxycorticosterone (DOC) are also considered as “neuroactive” because of their ability to modulate neurotransmitter activity (11). High density of the androgen receptors in regions responsible for learning and memory, such as hippocampus, suggests that there must be a relationship between the androgen receptors and cognitive function (12). Androgen receptors were also found in high density in mPFC which may mediate spine synapse formation in the mPFC of castrated male rats following treatment with androgens (13). Important reciprocal relationships between brain steroid hormone and neurotransmitter systems such as cholinergic (14), dopaminergic (15), GABAergic, serotonergic, and glutaminergic systems have been demonstrated (16). 

For instance, the modulatory activity of neuroactive steroids on the GABA system is well-known (11). GABA plays a controlling role in the balance of excitability and inhibitory states in the cortex and hippocampus (17-19). GABA_A_ is the main GABA receptors in the CNS targeted by substances with amnestic properties such as benzodiazepines and zolpidem (20). GABA_A_ receptors composed of five subunits that can belong to different subunit classes. The existence of six α, three β, three γ, one δ, one ε, one ө, one π and three ρ subunits gives rise to an enormous diversity of GABA_A_ receptor subtypes with different subunit composition and different pharmacological properties. The majority of GABA_A_ receptors; however, is composed of two α, two β, and one γ subunit (21). GABA_A_ receptor mediated signaling can be divided into two components. The first has been termed ‘phasic’ inhibition, (22) in this line, GABA_A_ receptors are the primary mediators of fast synaptic inhibition within the frontal lobes (8). A second form of GABAergic inhibition consists of continuous receptor activity, and is therefore known as ‘tonic’ inhibition. It changes only slowly with the ambient neurotransmitter concentration and may be critical for analog information processing (22, 23). Tonically active GABA_A_Rs can have profound effects on neuronal excitability, synaptic plasticity, network oscillations, and neurogenesis and have also been implicated in neuronal development, information processing, cognition, and memory (24). Neuronal activity in the cortex may also be regulated by tonic inhibition (25) which suggests a more important role in tonic inhibition in modulating mPFC pyramidal cell activity than that previously thought (26). Tonic inhibition via αβδ GABA_A_Rs has been shown to have an important role in shaping mPFC output (27). In this regard, GABA_A_ receptor containing δ subunit has been proposed to mediate behavioral responses to neurosteroids in the PFC (28). Sex steroids can influence neural activity indirectly by increasing the binding affinity of neurotransmitters or by directly altering cell membrane ion conductance in brain structures (16). Neurosteroids can be positive and negative endogenous modulators of GABA_A_ receptors (29). For instance, androstanediol has positive allosteric activity (9) and DHEAS has negative allosteric activity on GABA_A_ receptor (30). The modulatory activity of neuroactive steroids involves interaction with post-synaptic and extra-synaptic GABA_A_ receptors. Neuroactive steroids can also regulate the expression of GABA_A_ receptor subunit genes *in-vit*ro and *in-vivo*. As a consequence of these properties, allopregnanolone and the other neuroactive compounds modulate memory processes and may influence cognitive and neuropsychiatric symptoms such as those seen in AD (11). Testosterone appears to exert little regulatory control over GABA_A_ receptor subunit mRNA levels (31). A number of reports suggest that removal of the influence of inhibitory GABA receptors leads to memory enhancement and conversely the activation leads to memory inhibition. However, other results have reported the opposite, where GABA_A_ antagonists’ bicuculline injected into the hippocampus causes decrease in spatial memory (32). Also, the blockade of prefrontal cortex (PFC) GABA_A_ receptors with bicuculline impairs visuospatial attention in rats (33). The prelimbic mPFC contains pyramidal glutamatergic neurons that are modulated by GABAergic interneurons (34, 35). The prefrontal GABA hypofunction severely disrupts spatial reference and short term memory (36). Exogenous testosterone decreases plasma levels of both gonadotropins (37) and androgen preceursors such as dehydroepiandrosterone (DHEA) and its sulfate (DHEAS) (19, 38). Normal aging and cognitive dysfunction are associated with decreased levels of DHEA and DHEAS and therefore, provide tremendous opportunities for developing therapeutic approaches (39). Considering the data given above, we conducted a series of experiments to investigate the association between testosterone and GABA_A_ receptors, and their interaction on spatial learning and memory.

## Experimental

*Animal*

Male albino Wistar rats weighing 200–250 g, obtained from the breeding colony of the Pasteur Institute, were housed four per cage in a temperature and light-controlled room under a 12 h :12 h light:dark cycle, with water and food provided ad libitum (19). The experiments were performed on a 5-day protocol. 

*Surgery*

Seven days prior to initiation of the behavioral experiments, the rats were anaesthetized with a mixture of ketamine and xylazine (3 mg/100 g and 1 mg/100 g of body weight , respectively, i.p.) and two guide Cannulae were implanted bilaterally above the PL region of medial prefrontal cortex at coordinates: AP = +3.4mm (anterior to bregma), ML = ± 0.7mm from bregma (lateral to the midline) and DV = –3mm from dura (ventral to outer skull surface) based on the Paxinos and Watson’s atlas (36).

*Drug*s

The following drugs were used in this study, Testosterone purchased from Aburaihan pharmaceutical Company, Tehran, Iran. Bicuculline purchased from Sigma-Aldrich, St. Louis, MO, USA.

*Microinjection Procedure*

Drugs and vehicle were administered intra PL region through guide Cannulae (23-gauge) using injection needles (30-gauge) connected by a polyethylene tube to a 10 μL Hamilton microsyringe; the injection needle was inserted 0.5mm beyond the tip of the cannula and different doses of testosterone (0, 40, 80 μg, 0.5 μL DMSO/side; 30 min before training) or bicuculline (0, 2, 4 μg, 0.5 μL DMSO/side; 5min before training) (16) alone and co-treatment of testosterone (80 μg, 0.5 μL DMSO/side; 20 min before bicuculline injection) with bicuculline (2 μg, 0.5 μL DMSO/side; 5 min before training) were injected (pre-training) over 3 min. All injections (0.5 μL total volume) were delivered within two minutes with a syringe pump, and in order to avoid backflow the needles were left in place, an additional minute, before they were slowly withdrawn.

*Behavioral Assessment Apparatus*

The Morris Water Maze task consisted of a dark circular pool, 155 cm diameter and 55 cm height, filled with water (20 ± 1 ºC) to a depth of 25 cm. A transparent Plexiglas platform (11 cm in diameter) was located 1cm below the water surface in the center of one of the arbitrarily designed north-east (NE), south-east (SE), south-west (SW), or north-west (NW) orthogonal quadrants. The platform located in the SE quadrant provided the only escape from the water. Extra maze cues such as window, a door, shelves, and pictures on the walls surrounded the environment; the room was dark during the experiment, where the water maze was placed. These were kept in fixed positions with respect to the swimming pool, to allow the rat to locate the escape platform, hidden below the water surface. A PC computer with Ethovision (version XT7, Netherland) software, a CCD camera (Panasonic Inc., Japan) hanging from the ceiling above the MWM apparatus for monitoring and recording the position and movements of the rats in the water maze and a video tracking system for automated analyzing of the animal’s behavior were used. Thus, the time required reaching the platform (Escape latency), the swimming path (Traveled distance), and the swimming speed were recorded as well as the time and distance spent in each quadrant (19).

*Hidden Platform Trials*

All the rats were given a daily session of four trials for four consecutive days. Each trial involved placing the rat in the pool, close to and facing the wall in one of the four equal quadrants (north, east, south, and west). The animals were allowed to swim freely until they found the hidden platform located in target quadrant of the maze. If a rat failed to find the platform within 90s, the experimenter directed it toward the platform. After mounting the platform, the animals were allowed to remain there for 20 s. The rat was taken directly from the platform to the new starting point which was changed from trial to trial in random order; hence, each starting point was used once in each session of four trials. After completion of training, the animals returned to their home cages.

*Probe Test*

For the retrieval learning and memory test on the fifth day, in the probe trial the hidden platform was removed and the animal was released from the opposite location and allowed to swim freely for 60 s.

*Visual Platform Test*

After the probe trial, to assess visualmotor coordination, the platform was elevated above the water surface for being visible and placed in the different position (NE quadrant). The animals were released into the water from four different directions of the tank for four trials.

*Histology*

After the final test session, the rats were sacrificed using CO2. The brains were removed and fixed in a 10% formalin solution for 3 days and then in a 3% formalin solution at least 4 days. For histological examination of cannulae and needle placement in the PL region, 100μm thick sections were taken, and the cannula track was examined for each rat. We did not use the data from rats, in which cannula tips deviated from the target. 

In the remaining animals, all cannulae tips were just above the PL region and the injection tracks were exactly spread in a limited area, in the PL region of prefrontal cortex 

(Figure 1*).

*Statistical Analysis*

Data were expressed as mean ± SEM and processed by commercially available software SPSS 23. The data were analyzed using one-way analysis of variance and (RM) two-way analysis of variance (ANOVA) followed by Tukey’s post test, Bonferroni’s post test, and *T*- tests, and also, *P* < 0.05 was considered statistically significant for all comparisons. The charts are plotted by Graph Pad Prism 5.0 software.

*Experiments*

*Experiment 1*

The aim of this experiment was to determine the effect of pre-training bilateral injection of DMSO 30 min (testosterone vehicle), DMSO 5 min (bicuculline vehicle) and combination of both DMSO 5 min plus DMSO 30 min (testosterone vehicle + bicuculline vehicle) into PL region on spatial learning and memory. A total of 24 rats were divided into three groups, DMSO 30 group was treated by 30 min before training every day and DMSO 5 group was microinjected 5 min before training every day. DMSO 5 plus DMSO 30 group was treated by DMSO 30 min and 5 min before training. 

*Experiment 2*

The aim of this experiment was to determine the effect of bilateral pre-training injection of testosterone into PL region on spatial learning and memory. A total of 16 rats divided into two groups; 40 and 80 μg testosterone dissolved in 0.5 μL testosterone vehicle and microinjected 30 min before training every day. 

*Experiment 3*

The aim of this experiment was to determine the effect of bilateral pre-training injection of bicuculline into PL region on spatial learning and memory. A total of 16 rats divided into two groups; 2 and 4 μg bicuculline dissolved in 0.5 μL bicuculline vehicle and microinjected 5 min before training every day.

*Experiment 4*

The aim of this experiment was to determine the effect of bilateral pre-training injection of testosterone plus bicuculline into PL region on spatial learning and memory. For this purpose, 8 rats were treated by the effective dose of testosterone 80 μg, 30 min before and bicuculline 2 μg 5 min before training every day.

## Results

*Microinjection of DMSO into the PL did not affect spatial learning and memory performance*

The results obtained from the injection of DMSO showed no difference among DMSO 30 min (testosterone vehicle), DMSO 5 min (bicuculline vehicle) and combination of both (testosterone vehicle + bicuculline vehicle) prior to training in the spatial learning and memory performance. Also there is no significant difference between sham operated groups (vehicle groups) and control (data not shown). 

*Microinjection of testosterone into the PL induced spatial learning and memory impairment*

Figure 1 shows the results obtained from the injection of testosterone and its vehicle (DMSO 30 min). Analysis revealed a significant effect of treatment [*F* (2, 18) = 10.60; *P* < 0.0001] and a significant interaction between treatment and day [*F *(6, 54) = 3.002; *P *= 0.0132].

There was a significant increase in escape latency in the 1st (*P* < 0.01), 3^rd^, and 4th (*P* < 0.05) days in testosterone treated animals compared to the sham operated group and a significant increase in 1st (*P* < 0.001) day in testosterone 80 treated animals compared to testosterone 40 treated animals group (Figure 1A)., Also analysis showed a significant effect of treatment [*F *(2,72) = 14. 09; *P* < 0.0001] and did not show a significant interaction between treatment and day [*F *(6,72) = 0.3588; *P* = 0.9025] for traveled distance parameter. Further analysis using Tukey’s post test revealed a significant increase in traveled distance in 1st and 4th (*P* < 0.01) and 2nd (*P* < 0.05) days in testosterone treated animals compared to the sham operated group (Figure 1B). Analysis did not show any significant effect of treatment [*F* (2, 18) = 8.667; *P* = 0.0023] and interaction of treatment and day [*F *(6, 54) = 0.4534; *P* = 0.8394] for swimming speed (Figure 1C) indicating that PL injection of drugs did not affect motor capability of the animals. 

As shown in Figure 2, analysis revealed a significant effect of treatment [*F* (2, 18) = 4.176; *P *= 0.032] and there was a significant decrease in time spent in the target quadrant in testosterone treated rats compared with the sham operated group. Also, there was a significant decrease in time spent in the target quadrant (*P *< 0.05) in testosterone 80 treated animals compared to sham operated group (Figure 2A). However, no significant difference of distance to platform was observed (*P *= 0.606) (Figure 2B) in animal receiving testosterone compared to the sham operated group. No significant difference of escape latency was found between testosterone treated and sham operated group. (*P* = 0.636) (Figure 2C). No significant difference of velocity (*P* = 0.716) (Figure 2D) was observed in the visible test among different groups. This finding suggests that spatial memory was impaired by testosterone treatment in the pre-limbic. Also, visible test shows that testosterone has no effect on motivational and visual activities. This experiment has been analyzed by the One-way ANOVA and the Tukey’s post test.

**Figure 1*:**
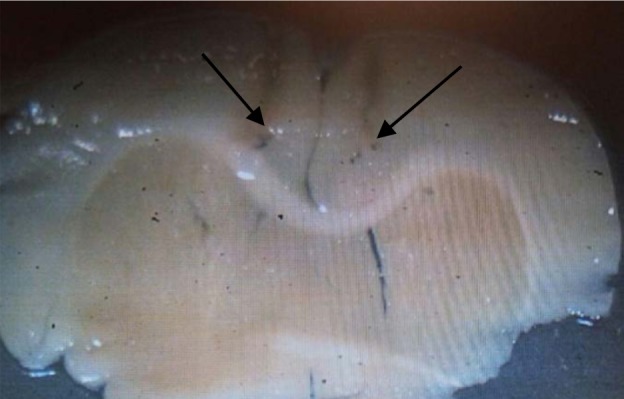
micrograph image of the injection site in the PL region

**Figure 1:**
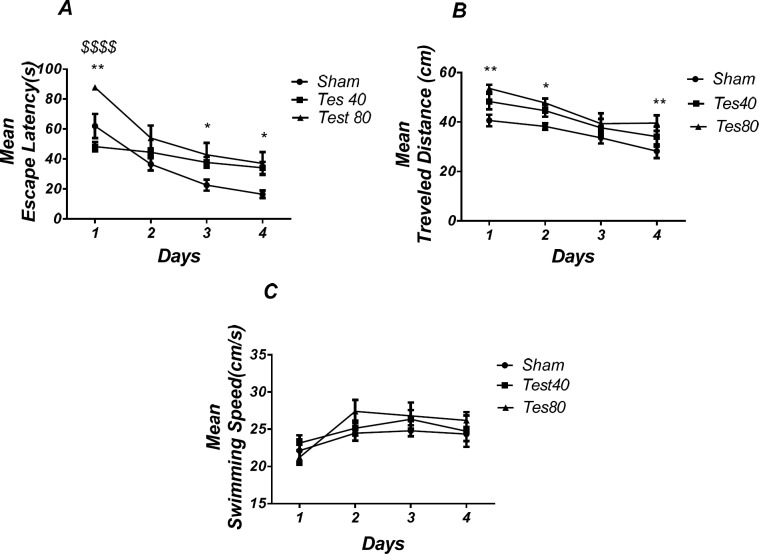
Effect of testosterone on spatial learning and memory**.** There was a significant increase in escape latency in1st (** *P* < 0.01), 3rd and 4th (* *P* < 0.05) days in testosterone treated animals compared to the sham operated group. Testosterone 80 treated animals showed a significant increase in 1st ($$$$ *P* < 0.001) day compared to the testosterone 40 treated animals group (A). Also, there was a significant increase in traveled distance in1st and 4th (* *P* < 0.05) and 2nd (* *P* < 0.05) days in testosterone treated animals compared to the sham operated group (B). No significant difference was found in swimming speed among different groups (C). (RM) Two-way analysis of variance (ANOVA) followed by post hoc analysis (Tukey test) were used and *P* < 0.05 was considered to be statistically significant. (n = 6-8 for each group)

**Figure 2:**
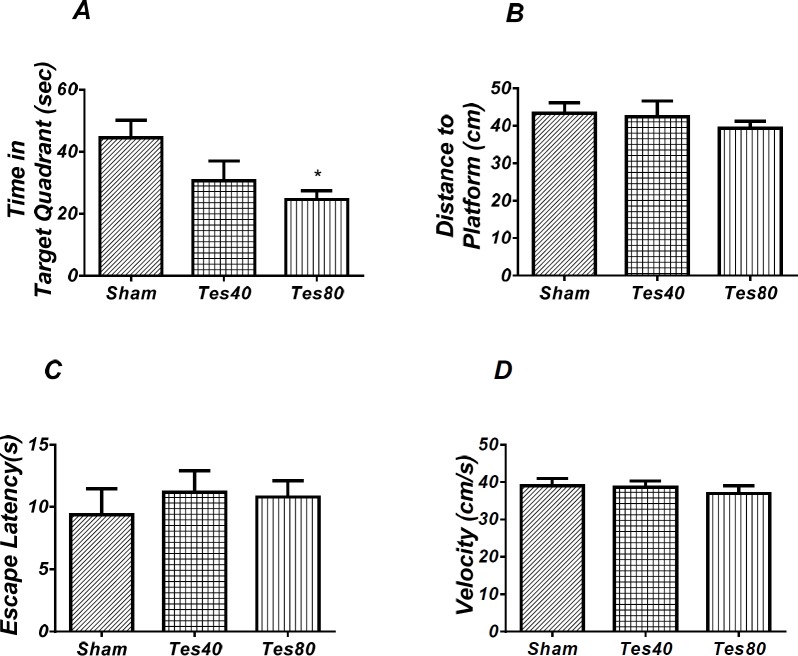
Effect of testosterone on probe test spatial learning and memory and visible test. There was significant decrease in time spent in the target quadrant in animal received testosterone 80 compared to the sham operated group (* *P* < 0.05) (A). There was no significant difference in distance to platform (B). In visible platform test there were no significant differences in escape latency (C) and velocity (D). One-way analysis of variance (ANOVA) followed by post hoc analysis (Tukey test) were used and *P* < 0.05 was considered to be statistically significant. (n = 6-8 for each group)

**Figure 3:**
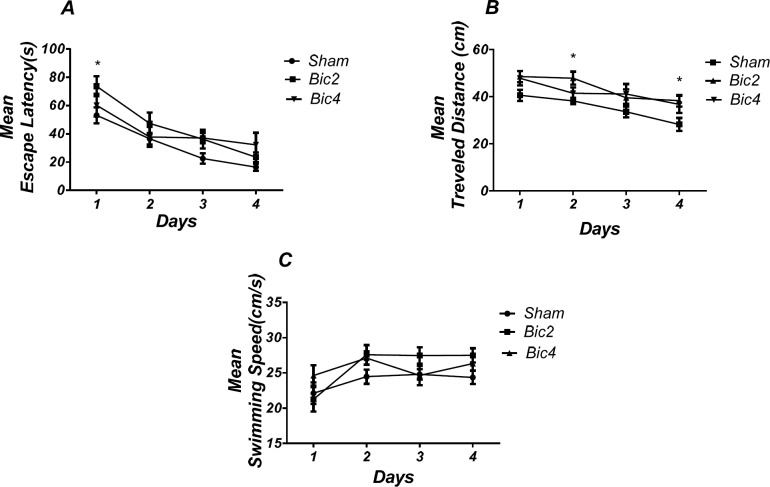
Effect of bicuculline on spatial learning and memory**.** There was a significant increase in escape latency in1st (* *P* < 0.05) day in bicuculline treated animals compared to the sham operated group (A). Also there was a significant increase in traveled distance in 2nd and 4th (* *P *< 0.05) days in bicuculline treated animals compared to the sham operated group. (B) No significant difference was observed in swimming speed among different groups (C). (RM) Two-way analysis of variance (ANOVA) followed by post hoc analysis (Tukey test) were used and *P* < 0.05 was considered to be statistically significant. (n = 6-8 for each group)

**Figure 4:**
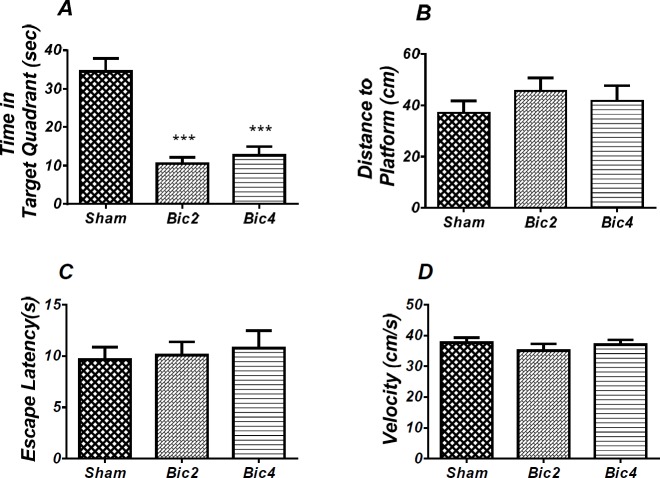
Effect of bicuculline on probe test spatial learning and memory and visible test. There was significant decrease in time spent in the target quadrant in animal received bicuculline compared to the sham operated group (*** *P*<0.001) (A). There was no significant difference in distance to platform (B). In visible platform test there were no significant differences in escape latency (C) and velocity (D). One-way analysis of variance (ANOVA) followed by post hoc analysis (Tukey test) were used and *p* < 0.05 was considered to be statistically significant. (n = 6-8 for each group)

**Figure 5:**
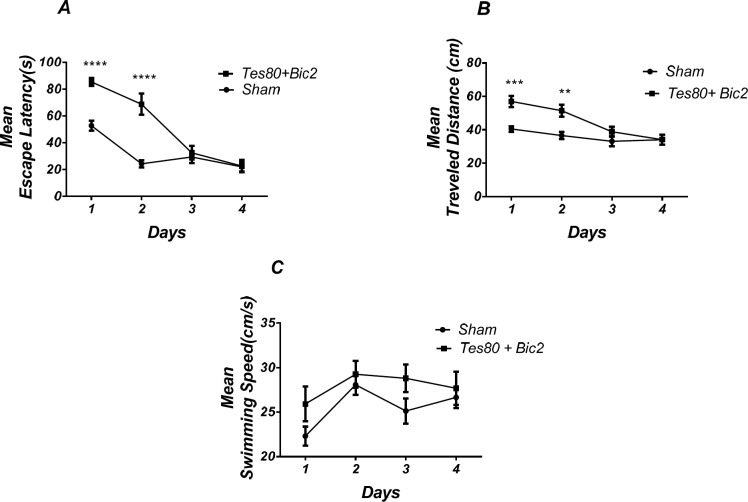
Effect of testosterone + bicuculline on spatial learning and memory**.** There was a significant increase in escape latency in1st and 2 nd (*** *P* < 0.001) days in testosterone + bicuculline treated animals compared to the sham operated group (A). Also, there was a significant increase in traveled distance in 1st (*** *P* < 0.001) and 2 nd (** *P* < 0.01) days in testosterone + bicuculline treated animals compared to the sham operated group (B). No significant difference was observed in swimming speed among groups (C). (RM) Two-way analysis of variance (ANOVA) followed by post hoc analysis (Bonferroni’s test) were used and *P* < 0.05 was considered to be statistically significant. (n = 6-8 for each group)

**Figure 6:**
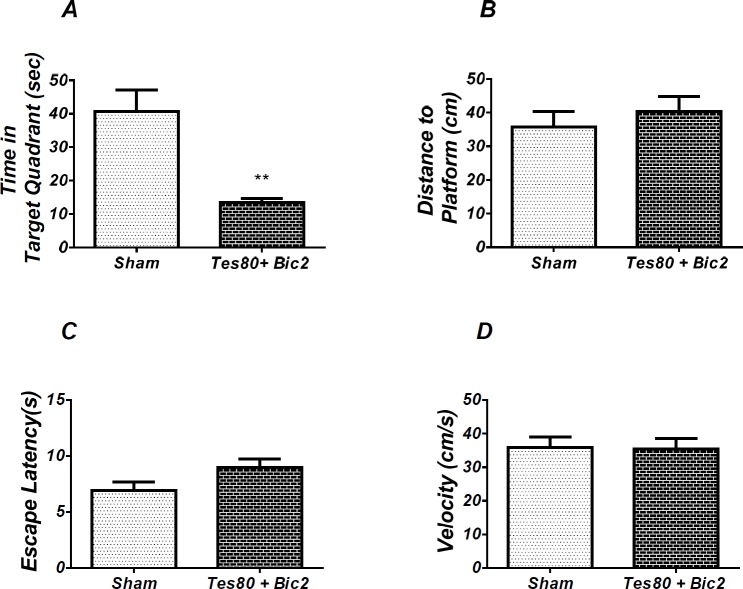
Effect of testosterone + bicuculline on probe test spatial learning and memory and visible test. There was significant decrease in time spent in the target quadrant in animal received testosterone + bicuculline compared to the sham operated group (** *P* < 0.01) (A). No significant difference was observed in the distance to platform (B). In visible platform test, there were no significant differences in escape latency (C) and velocity (D). *T*-test (un-paired) analysis was used and *P* < 0.05 was considered to be statistically significant. (n = 6-8 for each group)

**Figure 7:**
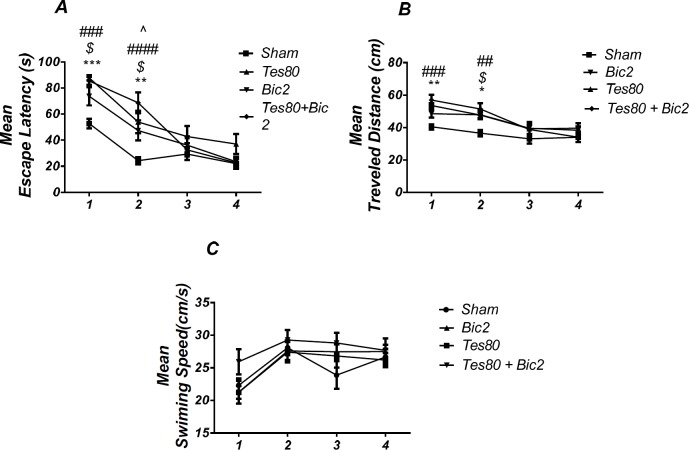
*Comparison* *of testosterone 80, bicuculline 2, testosterone 80 + bicuculline 2 and sham operated group*. There was a significant increase in escape latency in1st (*** *P* < 0.001) and 2 nd (** *P* < 0.01) days in testosterone treated animals, in 1st and 2 nd ($ *P* < 0.05) days in bicuculline treated animals, in 1st and 2 nd (### *P* < 0.001) days in testosterone + bicuculline treated animals compared to the sham operated group. There was a significant increase in escape latency in 2 nd (^ *P* < 0.05) day in the testosterone + bicuculline treated animals compared to the bicuculline treated animals group (A). Also, there was a significant increase in traveled distance in 1 st (** *P* < 0.01) and 2 nd (* *P* < 0.05) days in testosterone treated animals, in 2 nd ($ *P* < 0.05) day in bicuculline treated animals and in 1 st (### *P* < 0.001) and 2 nd (## *P* <0.01) days in testosterone + bicuculline treated animals compared to sham operated group (B). There was not a significant difference in swimming speed in days. There was not a significant difference in swimming speed in days (C). (RM) Two-way analysis of variance (ANOVA) followed by post hoc analysis (Tukey test) were used and *P* < 0.05 was considered to be statistically significant. (n = 6-8 for each group)

**Figure 8:**
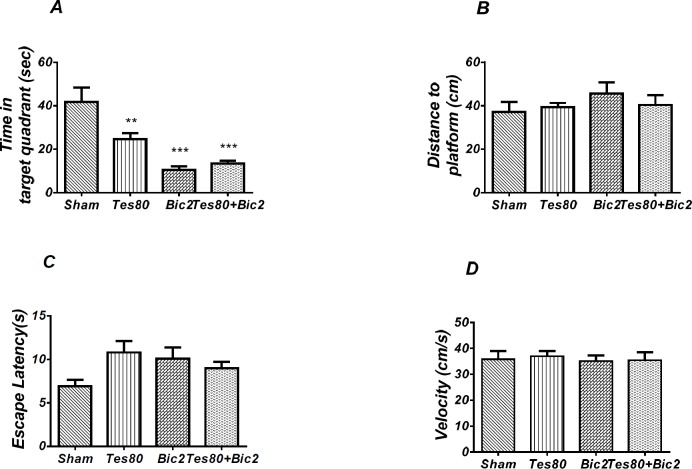
There was decrease significant in time spent in the target quadrant in animal received testosterone, bicuculline and testosterone + bicuculline compared to the sham operated group (** *P* < 0.01), (*** *P* < 0.001) (A) and there was no significant difference in distance to platform (B). In visible platform test there were no significant differences in escape latency (C) and velocity (D). One-way analysis of variance (ANOVA) followed by post hoc analysis (Tukey test) were used and *P* < 0.05 was considered to be statistically significant (n = 6-8 for each group)

*Microinjection of bicuculline into the PL induced spatial learning and memory impairment*

Figure 3 shows the results obtained from the injection of bicuculline and its vehicle (DMSO 5 min). (RM) Two way ANOVA analysis did not show any significant effect of treatment [*F *(2, 19) = 2.733; *P *= 0.0905] and interaction of treatment and day [*F* (6, 57) = 1.331; *P *= 0.2584] for escape latency. Further analysis using Tukey’s post test revealed a significant increase in escape latency in 1st (*P* < 0.05) day in the bicuculline 2 treated animals compared to the sham operated group (Figure 3A). Also, analysis of traveled distance showed a significant effect of treatment [*F* (2, 17) = 4. 673; *P *= 0.0241] and did not show a significant interaction between treatment and day [*F* (6, 51) = 0.7728; *P*= 0.5949] (Figure 3B). 

There was a significant increase in traveled distance in 2nd and 4th (*P* < 0.05) days in bicuculline treated animals compared to the sham operated group. No significant difference was observed in swimming speed between groups (Figure 3C).

As shown in Figure 4, analysis revealed a significant effect of treatment [*F *(2, 17) = 24.952; *P* = 0.000] and there was a significant decrease in time spent in the target quadrant in bicuculline treated animals (2 and 4 µg) compared to the sham operated group (Figure 4A). There was no significant difference in distance to platform (*P *= 0.502) (Figure 4B) in animal received bicuculline compared to the sham operated group. No significant difference was found in escape latency (*P* = 0.854) (Figure 4C) or velocity )*P* = 0.584( (Figure 4D) in the visible test between the groups. This finding suggests that spatial memory was impaired by bicuculline treatment in the pre-limbic. Also, visible test shows that bicuculline has no effect on motivational and visual activities. This experiment has been analyzed by the One-way (ANOVA) and the Tukey post test.

*Microinjection of combination of testosterone and bicuculline into the PL induced spatial learning and memory impairment*

Figure 5 shows the results obtained from the injection of testosterone + bicuculline. Analysis revealed a significant effect of treatment [*F* (1, 13) = 25.23; *P* = 0.0002] and a significant interaction between treatment and day [*F* (3, 39) = 14.05; *P* < 0.0001]. There was a significant increase in escape latency in the 1st and 2 nd (*P* < 0.0001) days in testosterone + bicuculline treated animals compared to the sham operated group (Figure. 5A). Also analysis showed a significant effect of treatment [*F* (1, 13) = 14; *P*= 0.0025] and a significant interaction between treatment and day [*F* (3, 39) = 4.608;* P* = 0.0075] for traveled distance parameter. Further analysis using Bonferroni’s post test revealed a significant increase in traveled distance in 1st (*P* < 0.001), 2 nd (*P* < 0.01) days in testosterone + bicuculline treated animals compared to the sham operated group (Fig. 5B) Analysis did not show any significant effect of treatment [*F* (1, 52) = 5.77; *P* = 0.0199] and did not show any significant interaction between treatment and day [*F* (3, 52) = 0.7266; *P *= 0.5407] for velocity (Figure 5C) indicating that PL injection of the drugs did not affect motor capability of animals.

As shown in Figure 6, there was significant decrease in time spent in the target quadrant in animal received testosterone + bicuculline compared to the sham operated group (*P *< 0.004) (Figure 4A). However, there was no significant difference in distance to platform (Figure 4B). There was no significant difference in escape latency (Figure 4C) or velocity (Figure 4D) in the visible test between the groups. This finding suggests that spatial memory was impaired by testosterone + bicuculline treatment in the pre-limbic. Also, visible test shows that testosterone along with bicuculline has no effect on motivational and visual activities. This experiment has been analyzed by the *T*- test (un-paired).

*Comparison of Sham Operated, Testosterone 80, Bicuculline 2 and Testosterone 80 + Bicuculline 2 groups*

Figure 7 shows the results from comparison of Testosterone 80, Bicuculline 2, Testosterone 80 + Bicuculline 2, and sham operated group (DMSO 30 min + DMSO 5 min). Analysis of escape latency revealed a significant effect of treatment [*F* (3, 26) = 7.359; *P* = 0.001] and a significant interaction between treatment and day [*F* (9, 78) = 3.545; *P *= 0.001]. There was a significant increase in escape latency in the 1st (*P* < 0.001) and 2 nd (*P* < 0.01) days in testosterone treated animals, in 1st and 2 nd (*P* < 0.05) days in bicuculline treated animals, in 1st and 2 nd (*P *< 0.001) days in testosterone + bicuculline treated animals compared to the sham operated group. There was a significant increase in escape latency in 2 nd (*P* < 0.05) day in testosterone + bicuculline treated animals compared to the bicuculline treated animals group (Figure 7A). Also, analysis of traveled distance revealed a significant effect of treatment [*F* (3, 24) = 6.962; *P* = 0.0016] but did not show a significant interaction between treatment and day [*F* (9, 72) = 1.769; *P* = 0.0891]. There was a significant increase in traveled distance in 1 st (*P* < 0.01) and 2 nd (*P* < 0.05) days in testosterone treated animals, in 2 nd (*P* < 0.05) day in bicuculline treated animals and in 1 st (*P* < 0.001) and 2 nd (*P* <0.01) days in testosterone + bicuculline treated animals compared to the sham operated group (Figure. 7B). Analysis did not show any significant effect of treatment [*F* (3, 24) = 1.506; *P* = 0.2383] and interaction of treatment and day [*F* (9, 72) = 0.9086; *P* = 0.5227] for swimming speed (Figure 7C). This experiment has been analyzed by the (RM) Two-way ANOVA and the Tukey post test. The post test multiple comparisons showed that microinjection of bicuculline after testosterone treatment did not reverse the spatial learning impairment when compared to the two groups used separately.

As shown in Figure. 8, there was a significant effect of treatment [*F* (3, 24) = 12.07; *P* = 0.000] and further analysis revealed a significant decrease in time spent in the target quadrant in testosterone 80 (*P* < 0.01), bicuculline 2(*P*< 0.001), and testosterone 80 + bicuculline 2 (*P* < 0.001) compared to the sham operated group (Figure 8A). However, there was no significant difference in distance to platform (*P *= 0.553) (Figure 8B) among different groups. There was no significant difference in escape latency (*P* = 0.092] (Figure. 8C) or velocity (*P* = 0.966) (Figure 8D) in the visible test between the groups. This test has been analyzed by the One-way ANOVA and the Tukey’s post test. The post test multiple comparisons showed that microinjection of bicuculline after testosterone treatment did not reverse the spatial memory impairment when compared to the two groups used separately. 

In other words, bicuculline could not rescue the spatial learning and memory deficit induced by testosterone. 

## Discussion

We found that microinjection of testosterone (80 µg), bicuculline (2 µg), and testosterone + bicuculline (80 +2 µg) in the prelimbic region displayed deficit in spatial learning and memory. It indicates that bicuculline could not improve learning and memory impairment induced by testosterone. Since there were no significant differences between the sham operated and experimental groups in visible platform performance, it can be inferred that the changes observed could not be attributed to alterations of non-mnemonic factors, such as the motivational or sensory processes induced by the treatments. Consistent with our results, chronic treatment with androgenic compounds impaired spatial learning and memory in adult animals (19, 38). Additionally, testosterone has been shown to cause dendritic spine loss in the hippocampus of mice (40). Also PFC GABA_A_ antagonist has been shown to impair spatial reference and working memory on a radial arm maze task (8). Additionally, the selective GABA_A_ receptor antagonist, bicuculline impaired acquisition, and consolidation in CA1 of hippocampus in the M W M task (19). There could be several possible explanations for our findings. 

First, in general steroids exhibit two different actions (21) and it has been proposed that there are two distinct sites for neurosteroids that act as positive modulators: one for allosteric enhancement of GABA, (41) in fact one site seems to be located within the four helical transmembrane domains of a subunit mediating allosteric modulation of receptors at low steroid concentrations (21) and another for direct activation of the receptor at high steroid concentrations that, in contrast, has been proposed to be due to steroid binding at a site on the interface between β and α subunits (41). This “GABA-mimetic” effect of neurosteroid is sufficient to suppress the excitatory neurotransmission (42). On the other hand, some studies showed the effects of bicuculline were completely reversible and indicate that it can both potentiate and antagonize GABA on the same cell, depending on the relative concentrations of the two substances (43, 44). In other words, bicuculline acts as a competitive antagonist at GABA_A_ receptors in that it competitively inhibits GABA binding to these receptors and, in turn, GABA competitively inhibits bicuculline binding (45). 

Therefore, testosterone as a GABA agonist causes impaired spatial learning and memory. Also testosterone is likely to increase the concentration of GABA in the environment. Consequently, GABA will compete with bicuculline as a competitive antagonist and prevent its effect. Therefore, in our study, testosterone may increase GABA high enough to blockade bicuculline effect on spatial learning and memory. 

Second, we know the prelimbic mPFC contains pyramidal glutamatergic neurons that are modulated by neurotransmitter systems such as GABAergic interneurons (34, 46). Testosterone, by acting as a non-selective sigma antagonist, may produce a tonic dampening of the function of sigma receptors and consequently decrease NMDA receptor function (low doses of testosterone did not modify the NMDA response). 

In this regard, it has been reported that testosterone, possibly via the AR, down regulated the NMDA receptor in CA1 in the rat (12, 47). One might speculate, since NMDA receptors are known to play an important role in spatial memory acquisition, such a decrease in NMDA receptor function induced by testosterone may be responsible for this observed decrease in spatial memory retention (47). Therefore, in our study, intra PL testosterone may involve glutamatergic system to induce spatial learning and memory impairment. 

Third, the modulatory activity of neuroactive steroids may be mediated through post- synaptic GABA-_A_ receptors, most commonly containing the α1 (GABRA1), β2 (GABRB2) and γ2 (GABRG2) subunits and extra-synaptic GABA_A_ receptors commonly containing α4 (GABRA4), σ (GABRD) or ε (GABRE) subunits (48). Neurosteroid modulation of GABAergic system plays a critical role in shaping both phasic and tonic synaptic inhibition in the CNS (29). However, depending on acting on γ-containing and δ-containing receptors the outcome may be different. 

The δ-containing receptors produce a much higher maximal response to neurosteroids (41) and they are highly sensitive to neurosteroids in certain brain regions (42). While several competitive, and non-competitive GABA_A_ receptor antagonists (gabazine, picrotoxin, and bicuculline) reduce phasic currents, they have no effect on tonic currents (42). In this line, while bicuculline as an antagonist is more effective in phasic inhibition, testosterone has a greater effect on tonic current and strengthens the GABAergic system. Thus, action of testosterone on tonic part while bicuculline blocks only phasic GABA inhibition may, in part, explain why bicuculline did not reverse the deteriorative effect of testosterone on learning and memory in our study. 

Fourth, previous studies have shown that GABA_A_R subtype expression profiles vary in different cortical layers (25). So, differences between superficial (II/III) and deeper layers (V/VI) were investigated (note there is no discernible layer IV in the mPFC) (49). In the superficial layers of the rodent PFC these GABA α4-, α2- and δ-subunits appear to be highly expressed (25, 50) and only α3, α5, and γ2 subunits are more highly expressed in the inner cortical layers compared to the superficial layers (41). Overall, δ-GABA_A_Rs tend to be more highly expressed in superficial layers (I and II/III) compared with the deep layers (layers V/VI) (42, 51) and it is responsible for tonic conductance in pyramidal cells (PCs) in layer II/III neocortex (27) and interneurons in the neocortex (52). Hence, the tonic inhibition in neurons from layer 5 is lower in magnitude compared to layer 2/3, likely due to the lower expression of the δ subunit in layer 5, (53) because the injection site was probably in the superficial layers, therefore, there are fewer receptors for bicuculline binding in this area, in contrast with testosterone that has more receptors in superficial layers. As a result, testosterone causes tonic currents and spatial learning and memory impairment.

Fifth, in some nuclei, there was a possibility that testosterone could be converted to estrogen by aromatized enzyme (12). Aromatase catalyzes the last step in estrogen biosynthesis (54). It has been aromatase causes to dendritic spine loss in the brain of mice, (12, 40, 55) hence impairing spatial memory (12). On the other hand, the results show that testosterone and estrogen reduce spatial learning and memory (56). In another study the seen estrogen in low and high concentrations reduces spatial performance (57) The increased expression of aromatase in the PFC was confirmed by immunohistochemistry (48) and changes in aromatase activity are also implicated in a wide range of human diseases, including Alzheimer′s disease. 

Animal studies suggest that brain aromatase activity is higher in adult males than in adult females and is modulated by changes in testosterone levels (54) Therefore, in our study, testosterone may have impaired spatial learning and memory through estrogen conversion.

Sixth, on the other hand, according to the comparison of thetestosterone, bicuculline and testosterone+bicuculline groups in the probe test, bicuculline increases the effect of testosterone and has synergistic effects.

## Conclusion

Our finding indicated that intra PL microinjection of testosterone and bicuculline separately impaired spatial learning and memory. Microinjection of bicuculline after testosterone treatment did not change spatial learning and memory impairment when compared to testosterone and bicuculline injected separately.
